# Intraoperative Maintenance of Normoglycemia with Insulin and Glucose Preserves Verbal Learning after Cardiac Surgery

**DOI:** 10.1371/journal.pone.0099661

**Published:** 2014-06-18

**Authors:** Thomas Schricker, Hiroaki Sato, Thomas Beaudry, Takumi Codere, Roupen Hatzakorzian, Jens C. Pruessner

**Affiliations:** 1 Department of Anesthesia, Faculty of Medicine, McGill University, Montreal, Quebec, Canada; 2 Department of Anesthesiology, Yamanashi University, Yamanashi, Japan; 3 Department of Psychiatry, Faculty of Medicine, McGill University, Montreal, Quebec, Canada; San Raffaele Scientific Institute, Italy

## Abstract

**Objective:**

The hyperglycemic response to surgery may be a risk factor for cognitive dysfunction. We hypothesize that strict maintenance of normoglycemia during cardiac surgery preserves postoperative cognitive function.

**Methods:**

As part of a larger randomized, single-blind, interventional efficacy study on the effects of hyperinsulinemic glucose control in cardiac surgery (NCT00524472), consenting patients were randomly assigned to receive combined administration of insulin and glucose, titrated to preserve normoglycemia (3.5–6.1 mmol L^−1^; experimental group), or standard metabolic care (blood glucose 3.5–10 mmol L^−1^; control group), during open heart surgery. The patients’ cognitive function was assessed during three home visits, approximately two weeks before the operation, and two months and seven months after surgery. The following tests were performed: Rey Auditory Verbal Learning Task (RAVLT for verbal learning and memory), Digit Span Task (working memory), Trail Making A & B (visuomotor tracking and attention), and the Word Pair Task (implicit memory). Questionnaires measuring specific traits known to affect cognitive performance, such as self-esteem, depression, chronic stress and social support, were also administered. The primary outcome was to assess the effect of hyperinsulinemic-normoglycemic clamp therapy versus standard therapy on specific cognitive parameters in patients receiving normoglycemic clamp, or standard metabolic care.

**Results:**

Twenty-six patients completed the study with 14 patients in the normoglycemia and 12 patients in the control group. Multiple analysis of covariance (MANCOVA) for the RAVLT showed a significant effect for the interaction of group by visit (*F* = 4.07, *p* = 0.035), and group by visit by recall (*F* = 2.21, *p* = 0.04). The differences occurred at the second and third visit. MANCOVA for the digit span task, trail making and word pair association test showed no significant effect.

**Conclusions:**

Preserving intraoperative normoglycemia by intravenous insulin and glucose may prevent the impairment of memory function, both short and long-term, after cardiac surgery.

## Introduction

Hyperglycemia is a typical feature of the body’s metabolic response to major surgical tissue trauma. During cardiac procedures this disturbance is severe with blood glucose levels exceeding 10 mmol L^−1^, even in the absence of diabetes mellitus [1]. Hyperglycemia is associated with poor outcomes after open heart surgery and postoperative cognitive dysfunction (POCD) [2]. While strict maintenance of normoglycemia reduces mortality in critically ill patients, there is only observational evidence to suggest benefits of tight glycemic control for cardiac surgical patients [3], [4]. This lack of evidence is due mainly to the fact that previous attempts to maintain glucose homeostasis during cardiac surgery failed, leading to the assumption that normoglycemia is not attainable [5]. The interaction between glucose metabolism, neuroendocrine and inflammatory changes induced by extracorporal circulation appears to be so complex that optimal glucose control is difficult to achieve.

In 2004 we introduced the ‘Glucose and Insulin administration while maintaining normoglycemia’ (GIN) concept in cardiac surgery [1]. In contrast to traditional insulin sliding scales this strategy modifies the rate of glucose infusion rather than changing the dose of insulin, which is kept constant. Using a preemptive insulin infusion followed by the administration of glucose at a variable rate we were able to keep blood glucose levels between 3.5 and 6.1 mmol L^−1^. In subsequent studies we demonstrated cardioprotective and anti-inflammatory effects of GIN in patients undergoing cardiac procedures [3].

Another functional domain that is compromised by open heart surgery is the integrity of the brain, in particular its cognitive function. There are reports suggesting that subtle neuropsychological deficits can still be detected one year after surgery [6].

Taking into account the relationship between hyperglycemia, inflammatory responses, functional alterations in the central nervous system and POCD, we tested the hypothesis that strict maintenance of normoglycemia with insulin and glucose (GIN), in contrast to standard metabolic care, preserves cognitive function after cardiac surgery, using four standardized cognitive measures, assessed three times over a period of seven months, as primary outcome measures. We found signs of impaired verbal memory in the standard care group at two and seven months post-surgery which was absent in the experimental group suggesting that maintenance of normoglycemia is indeed preserving cognitive function.

## Methods

Patients in this study participated in a larger registered Randomized Controlled Trial on the effect of intraoperative GIN therapy on outcomes after cardiac surgery (NCT005244) [7]. With approval from the McGill University Health Center Research Ethics Board, we approached patients scheduled for elective cardiac surgery and obtained written consent. Patients consented at least two weeks before their scheduled procedure. The inclusion criteria were ages 18–90 years and the ability to give written informed consent. Patients scheduled for off-pump coronary artery bypass grafting, with anticipated deep hypothermic circulatory arrest, elevated baseline troponin I levels (>0.5 ng L^−1^) or requiring hemodialysis were excluded. Sample size calculations assuming a medium effect size and employing the formulas provided by Cohen [16] suggested that we would need at least 20 subjects in each group to have a chance of at least 95% to find an effect in our sample if it was present in the population (λ = 11; power (1-beta) = .95).

### Study Protocol

Consenting patients were randomly assigned to the GIN group or the control group on the day of the surgery with the help of an assistant and by using a centralized computer system assigning patients to groups in all participating facilities. All surgeries took place in the morning hours of the day. Prior to induction of anesthesia, a baseline blood glucose value was obtained. Blood glucose concentrations were analyzed using the Accu-chek glucose monitor (Roche Diagnostics, Switzerland).

In the GIN group a priming bolus of 2 U insulin was followed by an infusion of insulin at 5 mU Kg^−1 ^min^−1^ (Humulin R regular insulin 100 U 100 mL^−1^ 0.9% normal saline) as described previously [1], [3]. Approximately 10 minutes after starting the insulin infusion, and when the blood glucose was <6.1 mmol L^−1^, dextrose 20% was administered intravenously. In the operating theatre, blood glucose levels were measured every 5–15 minutes and appropriate adjustments of the dextrose infusion rate were made to maintain the blood glucose within the target level of 3.5–6.1 mmol L^−1^. Table 1 shows the insulin adjustments made depending on blood glucose levels in the GIN group.

**Table 1:** Insulin adjustments depending on blood glucose levels.

If blood glucose (mmol L−1)	Action
>10.0	Increase insulin infusion by 3 U h−1
8.0–8.9	Increase insulin infusion by 2 U h−1
6.2–7.9	Increase insulin infusion by 1 U h−1
3.5–6.1	Maintain current insulin infusion rate
<3.5	Stop insulin infusion and administer a 10 mL dextrose 20%

In the control group, arterial blood glucose measurements were performed every 30 to 60 minutes while in the operating room. At any of these measurements, if the blood glucose was greater than 10.0 mmol L^−1^, an insulin bolus of 2 U was given followed by an infusion of 2 U h^−1^ (Humulin R regular insulin 100 U 100 mL^−1^ 0.9% normal saline). The insulin infusion rate was adjusted, according to the following sliding scale, to a maximum of 20 U h^−1^.

Postoperative glycemic control in both groups was performed using standard protocols aimed at a blood glucose concentration between 4 and 10 mmol L^−1^.

### Anesthetic and Surgical Care

All patients were anesthetized by the same two staff anesthesiologists. Patients received standardized total intravenous anesthesia using sufentanil, midazolam and pancuronium. Prior to cardiopulmonary bypass (CPB), heparin 400 IU Kg^−1^ was administered intravenously followed by additional doses, if necessary, to maintain an activating clotting time greater than 500 seconds. Protamine was administered as 1 mg 100 IU^−1^ of the heparin dose after complete separation from CPB.

Cardiopulmonary bypass was conducted with a roller pump and membrane oxygenator primed with a solution of 1 L of Ringer’s lactate, 5000 IU of heparin, 750 mL of Pentaspan (DuPont Pharmaceuticals Co, Newark, DE), and 44 mmol of bicarbonate. During CPB, pump flow was set at 2.4 times the body surface area, and mean arterial pressure maintained between 50 and 60 mmHg. The temperature was allowed to drift with active rewarming at the end of CPB. Cardioplegia solution (Plegisol, Hospira, Inc, Lake Forest, IL), used at the discretion of the cardiac surgeon, was free of glucose and consisted of high-dose (100 mmol L^−1^) or low-dose (40 mmol L^−1^) potassium.

### Cognitive Assessment

Cognitive function was assessed during three home visits, at approximately (a) two weeks before the operation (mean 15 days, range 1 to 90 days), (b) two months after surgery (mean 56 days, range 35 to 104 days), and (c) seven months after surgery (mean 204 days, range 180 to 254 days). The timing of the three home visits was not significantly different between the two groups (all *t*<1, *p*>0.20). Home visit schedule variation was caused by variations in the patients’ availabilities.

In line with recommended cognitive assessments after cardiac surgery [8], we employed the following tests:

#### (1) The Rey Auditory Verbal Learning Task (RAVLT)

This test measures verbal learning, memory, including proactive inhibition, retroactive inhibition, and retention. It consists of five presentations and immediate free recall of a 15-word list (List A), followed by an immediate free recall of a second presented word list (List B), followed by a sixth recall trial of List A. Delayed recall is then examined with a seventh recall of List A after a 20 minute delay without previous presentation. [9] The immediate and delayed recall of List A were used for statistical analysis.

#### (2) Digit Span Task

This task is used to assess working memory, and has two components. In the forward component, the subject is asked to repeat progressively longer lists of numbers after hearing them once. In the backward component, the subject must recite the number sequence backwards after listening to it once [10].

#### (3) Trail Making A & B

This task assesses visuomotor tracking and attention. Subjects are asked to draw a path between numbered circles in the ascending order. Subjects completed two different trails in random order at each visit (Trail A and Trail B). Dependent variables are number of errors and completion time in seconds, for each trail [11].

#### (4) Word Pair Task

This is an implicit memory task. The subject is shown 24 pairs of words. Immediately after, the first word of each pair is shown again and the subject is asked to recall the pair. This is repeated after 20 minutes. The total number of correctly recalled word pairs can be used for statistical analysis. The total number can further be differentiated for associated and non-associated words [12].

Questionnaires measuring specific traits known to affect cognitive performance, such as self-esteem, depression, chronic stress and social support were also administered. The questionnaires used for this purpose were the Rosenberg Self-Esteem Inventory, the Beck Depression Index, the Trier Inventory for the Assessment of Chronic Stress, and the Lubben Social Network Scale [13]–[15].

### Statistical Analysis

The RAVLT produced seven scores per visit: five immediate recall, one recall after interference, and a final delayed recall score. This was repeated for a total of three visits. To investigate the effect of cardiac surgery and GIN on the preservation of verbal memory, we ran a three factor (group by visit by recall) mixed design MANCOVA with the total number of recalled words as dependent variable, the repeated recall and the visit as repeated measures, and the group (GIN versus control) as independent variable. Age and years of education were entered as covariates.

Digit span testing resulted in two scores per visit, one forward and one backward. Thus, we ran a three factor (group by visit by direction) mixed design MANCOVA with the digit span score as the dependent variable, the direction and the visit as a repeated measures, and the group (GIN versus control) as an independent variable. Age and years of education were entered as covariates.

The trail making produced four different scores per visit, the time (in seconds) subjects took for completion, and the number of errors, for each of the two trails. Thus, we ran two two factor (group by visit) mixed design MANCOVAs with the time in seconds as the dependent variable, the visit as the repeated measure, and the group (GIN versus control) as independent variable. Age and years of education were entered as covariates.

The word pair association test produced six different scores per visit, the number of total, non-associated, and associated words, immediately after presentation (a), and again twenty minutes after (b). Thus, we ran three three factor (group by visit by recall) mixed design MANCOVAs with the number of recalled words as the dependent variable, the recall (immediate versus delayed) and the visit as repeated measures, and the group (GIN versus control) as independent variable. Age and years of education were entered as covariates.

## Results

**Figure 1:**
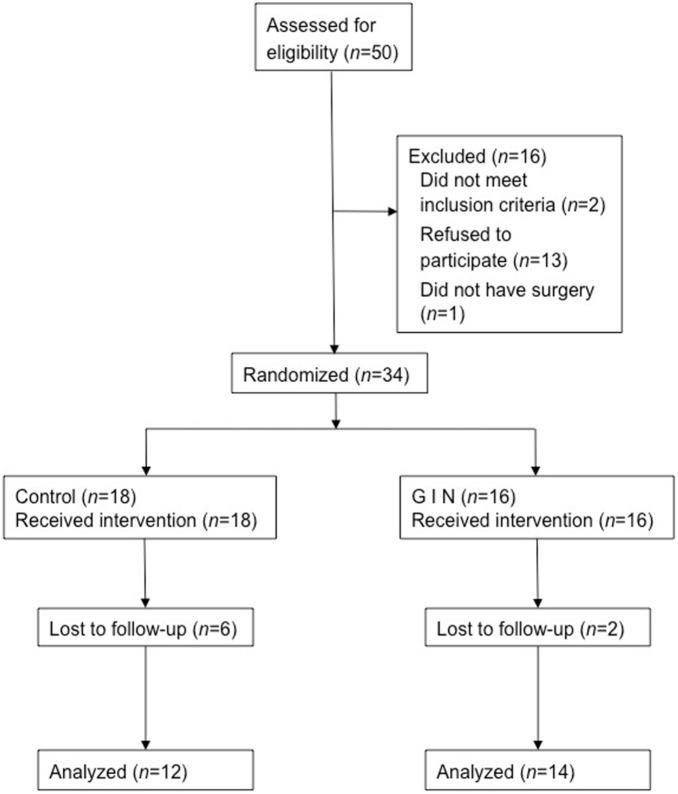
Schema illustrating the attrition in the GIN and control group over the course of the experiment.

Of the 50 patients approached 26 completed the study: 14 patients in the GIN and 12 in the control group (Figure 1). Patient characteristics were not significantly different from each other, and are presented in Table 2. Perioperative characteristics were also comparable in the two groups, and are shown in detail in Table 3. None of the patients suffered a serious adverse event. One patient in the therapy group had minor pneumonia, one patient a superficial wound infection and two patients had urinary tract infections. In the control group two patients were diagnosed with minor pneumonia and successfully treated. Two patients had urinary tract infections.

**Table 2:** Characteristics of patients.

Characteristic	Control	GIN^1^
Age ± SD (yr)	60±13	66±11
Gender (M/F)	9/3	10/4
Weight at admission ± SD (kg)	89±17	77±16
Height ± SD (cm)	172±8	171±9
Parsonnet score ± SD	12.2±8.2	16.5±9.3
Diabetes mellitus	4	5
HbA1c ± SD (%)	5.2±0.3	5.6±0.5
Creatinine ± SD (%)	90±22	95±25
Hematocrit ± SD (%)	40±4	42±8
Ejection fraction ± SD (%)	55±9	59±8

1 GIN = Glucose and insulin administration while maintaining normoglycemia.

**Table 3:** Perioperative data.

Characteristic		Control	GIN2
Type of surgery	CABG3	7	6
	Valve	3	4
	CABG and valve	2	4
Surgery time ± SD (min)		198±44	235±58
CPB4 time ± SD (min)		97±24	109±34
Cross clamp time ± SD (min)		75±24	91±31
Anesthesia time ± SD (min)		280±62	308±63
Intubation time ± SD (h)		6.3±6.9	8.2±5.5
Length of ICU5 stay ± SD (h)		22±8	21±10
Peak troponine ± SD (ng mL−1)		2.5±2.7	4.4±5.6
Peak lactate ± SD (mmol L−1)		2.5±1.4	2.0±1.5

2 GIN = Glucose and insulin administration while maintaining normoglycemia.

3 CABG = Coronary artery bypass grafting.

4 CPB = Cardiopulmonary bypass.

5 ICU = Intensive care unit.

All patients receiving GIN were normoglycemic during surgery. The mean blood glucose concentration in the control group was significantly higher (7.5±1.3 mmol L−1) than in the GIN group (5.0±0.5 mmol L−1, p<0.05).

**Figure 2:**
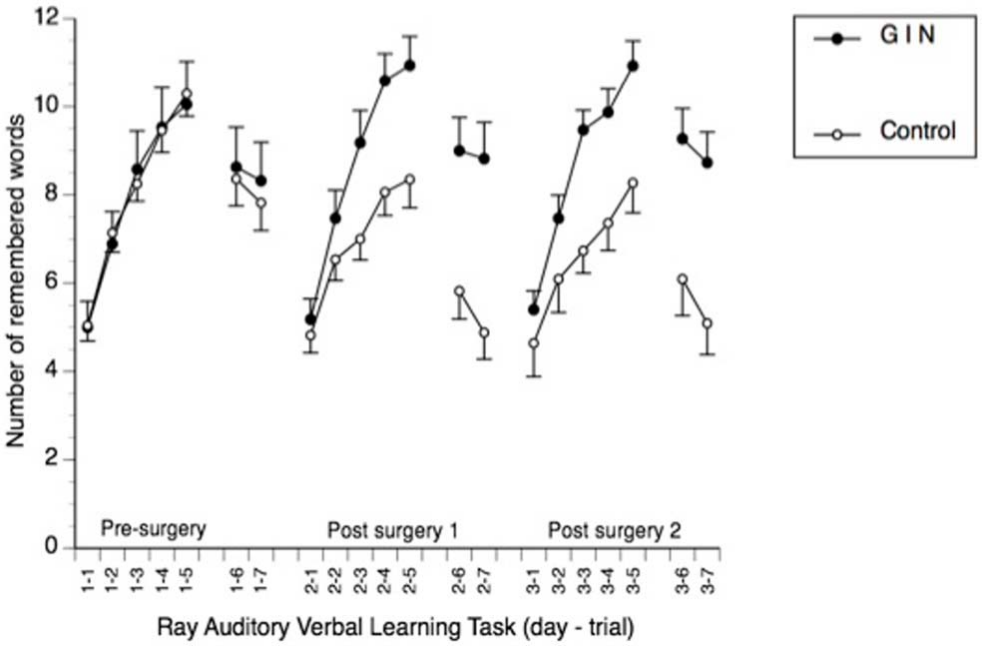
Performance of the subjects in the two groups over the course of the experiment. GIN: Experimental group with variable insulin infusion; Control: Best practice treatment; Pre-surgery: Neuropsychological assessment before surgery; Post surgery 1: Neuropsychological assessment approximately two months after surgery; Post surgery 2: Neuropsychological assessment approximately seven months after surgery; x-axis legend 1–1 to 3–7: days and iteration of verbal learning task.

The MANCOVA for the RAVLT showed no significant effect for group (*F* = 2.99, *p* = 0.12). As the assumptions of sphericity were violated, Greenhouse-Geisser corrections of the degrees of freedom for the interactions test were performed. Here, we found a significant effect for the interaction of group by visit (*F* = 4.07, *p* = 0.035), and group by visit by recall (*F* = 2.21, *p* = 0.04). There was a trend for the interaction effect of group by recall (*F* = 2.59, *p* = 0.07). Tukey *post hoc* tests revealed that the significant differences occurred at the second (recall three to seven) and third (recall two to seven) visit, but not on the first visit, suggesting a significant effect of the intervention on verbal learning (Figure 2).

For the digit span, the statistical analysis showed no main effect of group, no main effect of visit, and no significant interaction effects of group by visit, group by type, or group by type by visit (all *F*<1, *p*>0.20; see Table 4). There was a significant interaction effect of type by education (*F* = 8.8, *p*<0.02), with Tukey *post hoc* tests indicating that the subjects with higher education were performing significantly better in the reverse digit span.

**Table 4:** Raw scores and differences between groups for digit span, trail making and word-pair association tests over the three visits.

		GIN^2^		Control		
Test	Sub-test	Mean	SEM	Mean	SEM	Effect
**Digit Span**	1 forward	6.95	0.281	6.79	0.335	n.s.
	1 reverse	6.16	0.299	6.21	0.279	n.s.
	2 forward	7.35	0.296	7.18	0.439	n.s.
	2 reverse	6.59	0.285	6.04	0.284	n.s.
	3 forward	7.43	0.202	7.11	0.688	n.s.
	3 reverse	6.14	0.294	6.27	0.574	n.s.
**Trail making**	1a (secs)	108.39	15.466	101.88	11.343	n.s.
	1a (err)	0.11	0.111	0.28	0.158	n.s.
	1b (secs)	110.94	14.718	125.87	10.549	*p*<0.10
	1b (err)	2.5	0.984	5.13	3.3	n.s.
	2a (secs)	91.76	11.219	109.18	13.769	n.s.
	2a (err)	1.71	0.785	1.76	0.934	n.s.
	2b (secs)	97.71	12.168	133.24	18.008	*p*<0.05
	2b (err)	2.59	1.022	3.59	1.48	n.s.
	3a (secs)	99.07	8.168	126.27	15.165	*p*<0.05
	3a (err)	1.14	1.143	2.64	1.642	n.s.
	3b (secs)	90.21	10.394	139.09	26.503	*p*<0.05
	3b (err)	8.5	6.946	7.18	2.696	n.s.
**Word pair**	1a total	14.05	1.016	14.5	1.181	n.s.
	1a associated	12.53	0.762	12.89	0.882	n.s.
	1a non-associated	1.53	0.414	1.61	0.44	n.s.
	1b total	13.95	1.345	14.25	1.17	n.s.
	1b associated	12.68	1.095	12.93	0.938	n.s.
	1b non-associated	1.26	0.432	1.32	0.389	n.s.
	2a total	15	1.839	13.12	1.289	n.s.
	2a associated	12.71	1.254	11.88	1.018	n.s.
	2a non-associated	2.29	0.726	1.24	0.398	n.s.
	2b total	15.76	1.824	12.82	1.57	n.s.
	2b associated	13.41	1.21	11.41	1.176	n.s.
	2b non-associated	2.35	0.732	1.41	0.543	n.s.
	3a total	15.29	1.48	14.08	2.087	n.s.
	3a associated	13.07	1.112	12.55	1.598	n.s.
	3a non-associated	2.21	0.622	1.91	0.858	n.s.
	3b total	15.43	1.609	13.5	2.087	n.s.
	3b associated	13.07	1.174	11.82	1.56	n.s.
	3b non-associated	2.29	0.624	1.91	0.858	n.s.
**Age (yr)**		66.63	2.309	67.88	3.419	n.s.
Years of education		14.07	0.693	15.5	1.115	n.s.

2 GIN = Glucose and insulin administration while maintaining normoglycemia

For the trail making test, the analysis showed no main effect of group, no main effect of visit, and no significant interaction effects of group by visit, group by version, or group by version by visit (all *F*<1, *p*>0.20; Table 4). As previously, the effects were moderated by education (*F* = 6.2, *p*<0.02), and Tukey post hoc tests indicated that the subjects with higher education were performing significantly better in the trail making.

Finally, the statistical analysis of the word pair association test showed no main effect of group, no main effect of visit, and no significant interaction effects of group by visit, group by type, or group by type by visit (all *F*<2, *p*>0.15). No moderating effect of education was observed in this test either (Table 4). When comparing t-tests with the raw performance scores between the two groups, we observed differences that reached trend significance (*p*<0.10; Trail making 1b) or significance (Trail making 2b, 3a and 3b; Table 4). When correcting for multiple comparisons these trends were no longer significant, however.

From the significant effects found in the RAVLT, we calculated the effect size according to the formulas provided by Cohen [16]. Given an alpha-error of 0.05 and a sample size of 26, we estimated an effect size for the RAVLT result of *f* ^2^ = 0.09, resulting in an amount of explained variation between the groups of *ω* ^2^ = 0.08. In other words, about eight percent of the variation in the RAVLT scores between the groups across the visits was explained by the surgical procedure, after correcting for education and age.

## Discussion

The results of the present study suggest that strict maintenance of intraoperative normoglycemia, using intravenous insulin and glucose (GIN), contributes to prevent the impairment of both short and long-term memory function after open heart surgery. This benefit may be attributed to the preservation of normoglycemia, specific effects of insulin and/or the administration of glucose.

The pathophysiology of cognitive problems after cardiac surgery is complex and not well understood. Some evidence suggests that POCD is a consequence of temporary cerebral hypoxia *via* hypoperfusion and/or embolic events during the surgical insult. Because the ischemic brain is sensitive to increased circulating concentrations of glucose, intraoperative hyperglycemia and stimulated glucose uptake by neuronal cells may play a significant role [17]. Under anaerobic conditions intracellular glucose accumulation stimulates lactate production leading to lactic acidosis, protein glycosylation, generation of superoxide radicals and, ultimately, the conversion of ischemic penumbral regions into areas of frank infarction [18]. Hyperglycemia in the presence of cerebral ischemia augments the production and release of excitatory amino acid neurotransmitters, such as glutamate and aspartate, key mediators of ischemic cascades in the brain [17]. Furthermore, hyperglycemia enhances inflammatory responses as reflected by increased plasma concentrations of C-reactive protein, cortisol and cytokines which, by themselves, are neurotoxic [19].

While some authors propose that glycometabolic control is responsible for the beneficial effects of insulin, other studies indicate that insulin itself is important [20]. Insulin receptors in brain cells have been shown to be instrumental in regulating cognitive function, i.e. learning and memory [21]. Furthermore, insulin, especially when administered at higher doses as in the GIN protocol, exerts non-metabolic effects including vasodilatory, anti-inflammatory, anti-oxidative, anti-aggregatory, positive inotropic and cardioprotective effects [22]. Because insulin is a potent stimulator of endothelial nitric oxide formation and an inhibitor of tumor necrosis factor synthesis, it may ultimately promote neuron survival and reduce apoptosis [23]. Insulin resistance, the endocrine mechanism responsible for hyperglycemia in the context of surgery, has been linked to poor cognitive performance in nondiabetic subjects [24]. Low cognitive scores were observed in middle aged individuals with low insulin sensitivity [25]. Overcoming insulin resistance during surgery through exogenous administration of insulin can therefore be neuroprotective. This interaction between insulin resistance, cognitive dysfunction and positive effects of insulin administration on memory performance prompted some to suggest that Alzheimer’s disease could be considered a form of diabetes mellitus of the brain [26]. Conversely, the intranasal application of insulin has been demonstrated to improve cognitive function in patients with minimal impairment or overt Alzheimer’s disease [27].

Glucose ingestion *per se* could be the third mechanism underlying the positive influence of GIN on memory function after surgery. In humans, the strongest effects of glucose are observed in the elderly, subjects with dementia and poor glucose regulation [28]. Microinjections of glucose into the septohippocampal system of rats enhanced mnemonic function, may be mediated through an increased synthesis and release of acetylcholine. This assumption is supported by observations showing a modifying impact of glucose on neural and behavioral effects of cholinergic drugs.

Of note, all of the previously proposed mechanisms point at the hippocampal formation as a possible target and mediator. The significant effect of maintaining normoglycemia on a verbal learning paradigm further suggests that it might have been the left hippocampal region that benefitted in particular from the experimental protocol, as language is typically associated with the left hemisphere, and verbal memory is associated with the left hippocampus. However, this interpretation has to be considered speculatory until further studies would allow us to also assess the integrity of selected structures in the central nervous system, for example by use of neuroimaging assessments.

Taken together, the results of this study provide preliminary evidence of neuroprotective effects of GIN during open heart surgery which appear to be specific to learning and memory and, thus, likely affect the hippocampus.

There are a number of limitations that need to be mentioned. First, the number of subjects was rather small for such a trial; unfortunately due to budget and time availability constraints of the involved personnel we were unable to test a larger number of patients. An amount of 8% of variability in the memory data explained by the intraoperative procedure indicates a small to medium effect size, suggesting that other factors (e.g., genes, lifestyle, diet, cognitive training, occupation etc.) contributed significantly to the variability of individual memory performance as well. Furthermore, the small number of tested subjects could lead to an overestimation of the treatment effect, with larger samples typically producing smaller effect sizes, and group differences. Nevertheless, the statistical analysis indicated that maintenance of normoglycemia was a significant factor in this particular type of memory performance, thus we consider these results as promising yet preliminary evidence for the effect of maintaining normoglycemia. Second, the groups were not very homogeneous; the type of cardiac surgery was not balanced between the two groups, and the age of the participants ranged from young to old adulthood, making the distribution of this variable rather heterogeneous within the groups. Due to the nature of the experimental setup (testing the effects of maintaining normoglycemia for any open heart surgery patients scheduled to occur during the testing period), this was unavoidable. However, the mean age was not different between the control and experimental group, and entering age as a covariate in the analyses did not affect the results. Third, it can be speculated whether the maintenance of normoglycemia might be especially beneficial for patients already at risk of cognitive decline, e.g. subjects with subjective memory complaints, or mild cognitive impairment. Since it can be assumed that the cognitive performance of subjects with these symptoms might have been significantly different at the onset, and would have likely shown a stronger decline over time, those subjects should then form separate groups. While this is a valid hypothesis that should be tested in future experiments, our current study did not allow to test for this given the low number of participants, and the heterogeneous study groups. Finally, while patients were controlled for perioperative blood pressure and no group differences were observed, it is possible that fluctuations in bisprectal index or cerebral oxymetry, which were not monitored in our institution, could have influenced postoperative cognitive function.
